# Remarks on the Case Related by S. J. E. in Page 18 of the Present Volume

**Published:** 1817-04

**Authors:** 


*For the London Medical and Physical Journal.*

### *Remarks on the Case related by S. J. E. in page* 18 *of the present Volume;* by J. A.

IT is evident, by the history, that the patient is subject to inflammatory complaints, which have probably increased in frequency since the cessation of menstruation, and since the irritability of her constitution has been increased from those causes which rendered attention to the wants of her family more necessary.

As far as this goes, the remedy is simple, if it can be complied with,—namely, that her mind should be kept as free as possible from anxiety; her diet simple; and frequent local bleeding, either by leeches or cupping glasses. Add to these, great care should be taken to keep the head as cool as possible, and the feet, with the whole lower extremities, as warm.

The next question is, Whether there is any thing like a periodical return of the paroxysm? If this return be regular, and, as would appear by one part of the account, with more or less violence most nights and part of the morning, Fowler's solution should be tried in the dose of five drops three times a day. If the periods are more distant, their probable return should be calculated with as much certainty as possible, either by the usual period of return, or by any symptoms which may have been observed to precede the paroxysm; and, at times, evacuations, local and general, should be used with great freedom, all the attendant and consequent symptoms and effects being watched with the greatest attention. In either case, the patient should drink nothing stronger than water or thin table-beer.

Such are all the opinions that the writer of the above feels himself authorized to give from the present, as he conceives, imperfect statement of the case.

